# Novel embeddings improve the prediction of risk perception

**DOI:** 10.1140/epjds/s13688-024-00478-x

**Published:** 2024-05-22

**Authors:** Zak Hussain, Rui Mata, Dirk U. Wulff

**Affiliations:** 1 Faculty of Psychology, University of Basel, Missionsstrasse 60–62, Basel, 4055 Switzerland; 2 Center for Adaptive Rationality, Max Planck Institute for Human Development, Lentzeallee 94, Berlin, 14195 Germany

**Keywords:** Risk perception, Language models, Psychometric paradigm, Free associations

## Abstract

We assess whether the classic psychometric paradigm of risk perception can be improved or supplanted by novel approaches relying on language embeddings. To this end, we introduce the Basel Risk Norms, a large data set covering 1004 distinct sources of risk (e.g., vaccination, nuclear energy, artificial intelligence) and compare the psychometric paradigm against novel text and free-association embeddings in predicting risk perception. We find that an ensemble model combining text and free association rivals the predictive accuracy of the psychometric paradigm, captures additional affect and frequency-related dimensions of risk perception not accounted for by the classic approach, and has greater range of applicability to real-world text data, such as news headlines. Overall, our results establish the ensemble of text and free-association embeddings as a promising new tool for researchers and policymakers to track real-world risk perception.

**Supplementary Information:**

The online version contains supplementary material available at 10.1140/epjds/s13688-024-00478-x.

## Introduction

Individuals and societies may be facing an increasingly large number of natural and technological risks [e.g., 1–3]. How these risks are perceived matters. Meta-analyses suggest that risk perception motivates the adoption of health behaviors [4], acceptance of novel technologies [5], and climate change adaptation behavior [6]. As such, being able to anticipate how people think about risk in the real world and communicate more effectively on the topic is of interest to researchers and policymakers alike.

A seminal approach to understanding risk perception is the psychometric paradigm [7, 8], which is characterized by its use of survey methods to identify psychological dimensions underlying people’s perception of risk. The paradigm traditionally elicits people’s judgements of risk and related dimensions numerically. These dimensions quantify how specific risks are perceived by measuring, for instance, how controllable the risk is thought to be, how much dread it evokes, or how fatal people think its consequences are. Individual judgements are often aggregated to obtain a mean estimate for each risk. One conclusion of this work is that risk perception can be mapped into a psychological space composed of (at least) two dimensions, often termed *dread* and *unknown* [8, 9].

Despite its prominence in the literature, the psychometric paradigm has a number of limitations when it comes to predicting risk perception. First, because it is resource-intensive to collect participant ratings, psychometric data sets tend to be limited in size, containing only a few hundred risks in their vocabulary. This is particularly problematic from a prediction perspective, where researchers may wish to generalize their models beyond the laboratory to more diverse linguistic environments such as digital media, which both reflect and arguably shape public risk perception [e.g., 10, 11]. Second, because the paradigm is based on a set of questions (or dimensions) that have been explicitly chosen or engineered by the researchers, it is possible that some relevant dimensions have escaped inclusion because they were never considered but are nonetheless relevant to the public’s risk perception [12].

Recent developments in machine learning and artificial intelligence have led to new tools that can help us overcome the limitations of the psychometric paradigm. In particular, these developments have made available so-called language embeddings that provide quantitative representations of the meaning of linguistic units (e.g., words) in a language in terms of high-dimensional numerical vectors. Embeddings are trained on vast quantities of domain-general digital text resulting in vocabularies in the order of millions of linguistic units. Research has shown that such embeddings can capture important aspects of the human cognitive system, including memory, reasoning, and judgment [e.g., 13, 14]. Embeddings have two key advantages over the psychometric paradigm. First, their vast vocabulary, which may effectively contain all linguistic stimuli in a given language, can, in principle, help evaluate any current and future risks represented in linguistic terms. Second, the numerical vectors are not limited to the dimensions chosen by researchers but instead encompass an array of abstract semantic features that implicitly include extant or novel features of risk perception.

Past work studying risk perception using embeddings has demonstrated that an early-generation neural network word embedding, commonly referred to as *Word2Vec*, could explain a considerable portion of risk perception variance. Although this model was generally outperformed by the psychometric paradigm [15], in recent years, newer and potentially more promising embeddings have become available that have been trained on more text and with improved architectures, such as “Global Vectors” (*GloVe*) [16] and *fastText* [17]. Furthermore, a new class of context-aware models known as transformers have also entered the scene [e.g., “Bidirectional Encoder Representations from Transformers” (*BERT*); 18, 19], which show impressive performance in predicting human behavior [14].

Although most embeddings are trained on written text, this is not the only and may not even be the single best source of information to capture people’s semantic representations [20, 21]. Another approach is to obtain embeddings from free association, a paradigm in which participants are given a cue word and asked to respond with one or more words that come to mind. Recently, free association data sets have become available that are large enough to train high-quality embeddings. For example, the *Small World of Words* (SWOW) citizen-science study aims to produce population-level semantic representations in several languages. The English SWOW project contains millions of responses to over 12,000 cue words [22]. Representations derived from free associations have been found to be a powerful contender to text embeddings when predicting human judgments and behavior [23–26], and have shown promise in elucidating group differences in the representation of risk [21]. This may be because text and free associations reflect differently factors that go beyond semantic relations, such as pragmatic communication rules [21, 24].

In this study, we evaluate whether novel embeddings can further improve our ability to predict and understand risk perception beyond the classic psychometric paradigm. We seek to address three main questions. First, we ask how well novel embeddings—specifically, more recent text and free-association embeddings—predict risk perception when compared to the classic psychometric approach. For this purpose, we introduce a novel data set—the Basel Risk Norms—capturing the largest set of risk sources and associated psychometric ratings to date. The large coverage allows us to adequately assess the relative performance of different models using cross-validation prediction methods. Second, we assess to what extent novel embeddings help reveal dimensions not currently accounted for by the psychometric approach. We address this issue by using a novel interpretability approach that assesses the nature of the unexplained variance remaining from the psychometric paradigm using interpretable dimensions of word norms, such as affect, concreteness, or frequency, and comparing this to ensemble models that integrate the psychometric paradigm with novel embeddings to assess which aspects are better captured by the latter. Finally, we assess the extent to which the classic psychometric paradigm and novel embeddings can be applied to predicting risk perception associated with real-world text, such as digital news, which is an important source of risk information. Specifically, we assess the relative coverage of the psychometric paradigm and the alternative novel embeddings to over 15,000 news headlines. All in all, we hope to contribute to clarifying how novel embeddings can enrich the toolbox of approaches used to predict real-world risk perception.

## Results

### Basel risk norms

Investigations of risk perception typically rely on data containing a few dozen [7] to, at most, a few hundred risks [15]. However, data of this magnitude are not ideal for evaluating the accuracy of prediction models of risk perception, especially given the large number of parameters that must be estimated for high-dimensional models involving language embeddings. To overcome this limitation, we generated a new data set of risk norms—the Basel Risk Norms—consisting of risk perception information concerning 1004 risk sources (e.g., vaccination, nuclear energy, artificial intelligence) and associated ratings on nine psychometric dimensions typically used in the literature (*Calm–Dread, Not-Fatal–Fatal, New–Old, Chronic–Catrastophic, Known–Unknown*) (see Table 1). The Basel Risk Norms present the largest and most reliable data set of risk perception to date, exceeding both the number of sources as well as associated reliabilities of the human ratings for risk and psychometric dimensions of past studies in the risk perception literature.

**Table 1:** Names and descriptions of the risk item and nine psychometric items used in the Basel Risk Norms and previous literature [e.g., 7, 9, 15]

Name	Item
Risk	How risky or safe is the following?
Voluntary–Involuntary	Are individuals exposed to this risk voluntarily or involuntarily?
Immediate–Delayed	Is death from this risk immediate or delayed?
Known–Unknown	Is this risk known or unknown to the individuals exposed to this risk?
Known–Unknown (Sci.)	Is this risk known or unknown to science?
Controllable–Uncontrollable	Is this risk controllable or uncontrollable for the individual exposed to the risk?
New–Old	Is this risk new or old?
Chronic–Catastrophic	Is this a risk that kills one person at a time (chronic) or a risk that kills large numbers of people at once (catastrophic)?
Calm–Dread	Is this a risk that individuals can reason about calmly or is it one that they have great dread for?
Not-fatal–Fatal	How fatal are the consequences of this risk?

**Figure 1:**
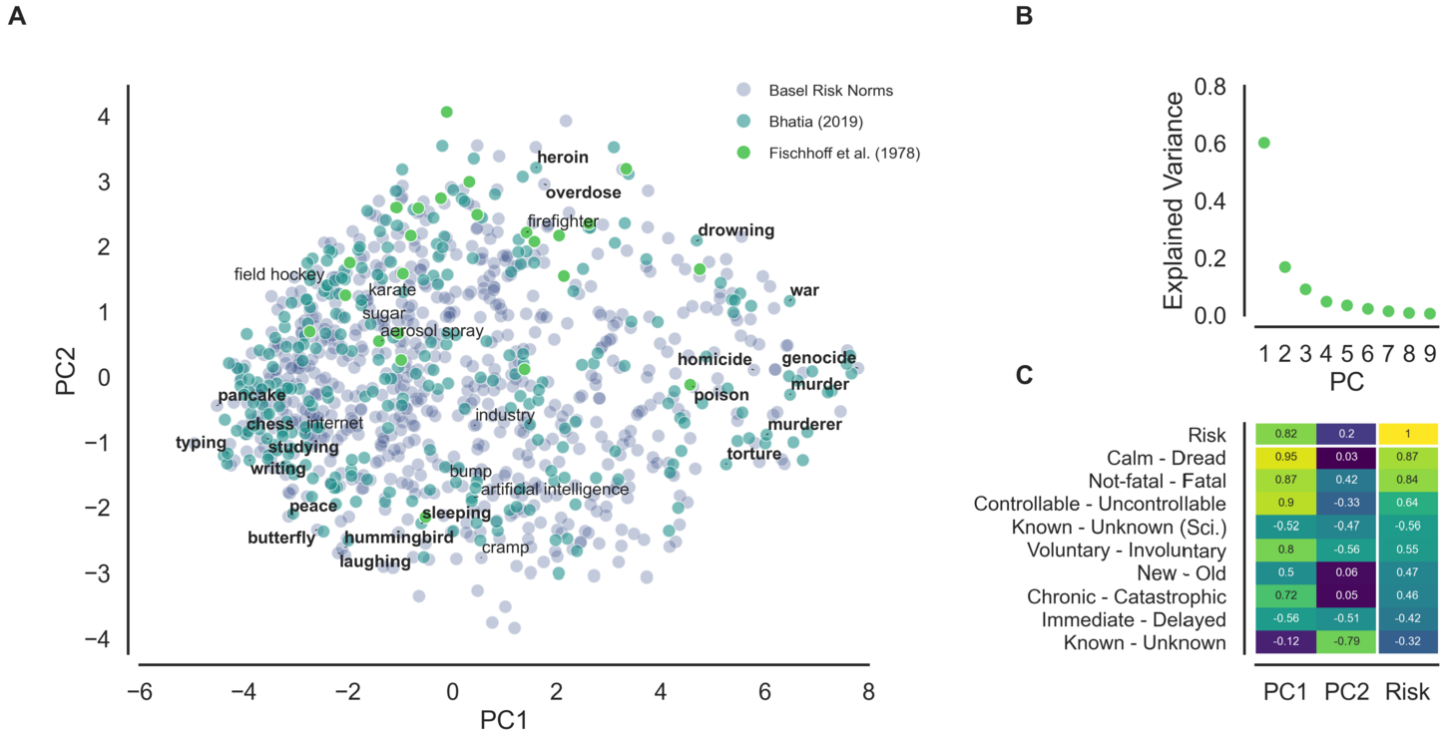
Overview of the Basel Risk Norms. **A.** 1004 risks according to the first two principal components of the psychometric ratings. 10 risks sampled from the top 20 riskiest (bold), 10 from the 20 safest (bold), and 10 from the mid-20 rated sources are annotated. **B.** Psychometric rating variance explained by each principal component. **C.** Pearson correlation of the first two principal components with each psychometric dimension, colored according to absolute correlations and ordered according to absolute correlation with the risk ratings

To give an overview of these data, and in line with past investigations of risk perception that summarize the data using dimensionality-reduction techniques [7, 15], we conducted a principal component analysis of the nine psychometric items. Several noteworthy insights emerged. First, consistent with previous findings [7, 8], two components (see Fig. 1A) accounted for the majority of the psychometric variance (almost 80%). However, this is predominantly due to the first principal component, with the second component explaining only marginally more than the third, fourth, or fifth component. A similar pattern can be observed for the relationship between the components and risk perception. Next to a highly correlated first component ($r=.82$), components two, three, five, six, and eight are also significantly related to risk perception, albeit less strongly. This suggests that, beyond a single central dimension, risk perception is a multidimensional construct.

Second, the first two components do not fully replicate the traditional *dread* and *unknown* factors of the psychometric model [7]. Although the first component effectively captures dimensions associated with *dread* (e.g., *Calm–Dread, Not-Fatal–Fatal*), the second does not, as one would predict, capture fully the dimensions associated with *uncertainty*, with some uncertainty-associated dimensions (e.g., *Known–Unknown* and *New–Old*) being equally or even more related to the first than the second component (see Fig. 1B). This pattern of findings suggests that the separation into two main components (dread, unknown) may oversimplify the structure of risk perception when considering a large(r) number of risks as done by the Basel Risk Norms.

Third, and finally, the additional risks included in our norm data set appear to fill gaps within the psychometric space not covered by previous data sets by [7] and [15], suggesting that the Basel Risk Norms cover the risk perception space with greater resolution than previous data collection efforts.

All in all, our newly generated data set represents the largest and most reliable data set of risks available to date. The data set aligns largely with recurring patterns in the literature on the psychometric paradigm but also reveals noteworthy deviations, suggesting that risk perception may involve multiple dimensions that go beyond the two proposed by the classic psychometric paradigm. In what follows, we further address the ability of the classic paradigm to capture the richness of this multidimensional representation and contrast it with a number of novel embeddings in predicting risk perception.

### Using novel embeddings to predict risk perception

**Figure 2:**
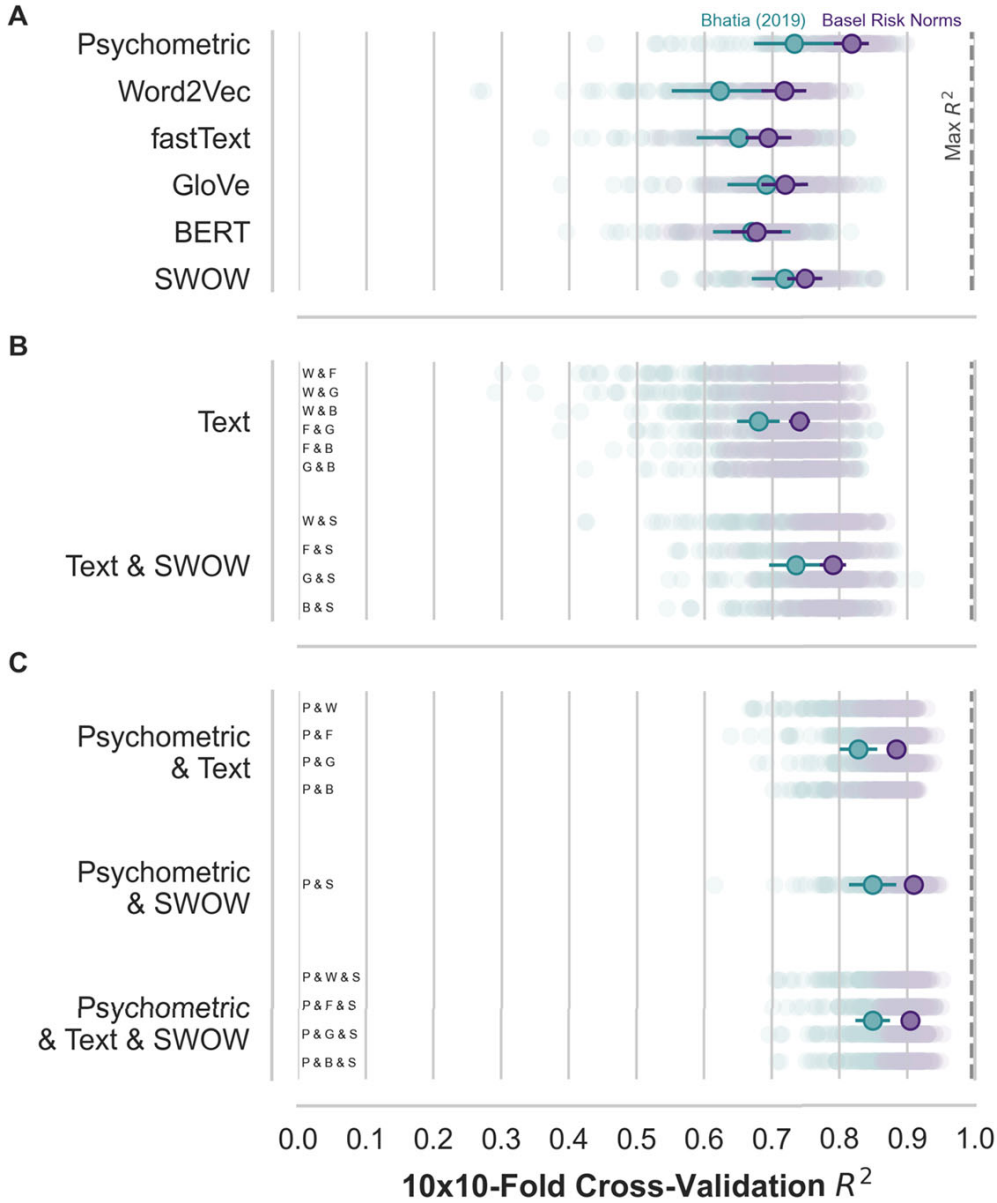
Prediction of risk perception. A model comparison using the Basel Risk Norms (1004 risks) and [15]’s data (306 risks). Foreground points are the (grand) means of the test set performances used in cross-validation (10×10-Fold) whereas background points reflect the individual test set performances. The Max $R^{2}$ (vertical dashed line) represents the reliability of the risk ratings. **A.** Individual model comparison. **B.** Text and SWOW Ensembles. **C** Psychometric, Text, and SWOW Ensembles. Error bars are adjusted 95% confidence intervals [27]. Model ensembles in **B.** and **C.** are labeled with letters representing the individual models composing them: P = Psychometric, W = Word2Vec, F = fastText, G = GloVe, B = BERT, S = SWOW

How do novel embeddings fare relative to the psychometric paradigm in predicting risk perception? To answer this question, we relied on the new Basel Risk Norms to compare different models’ ability to predict the average risk perception concerning 1004 risks, including, to name but a few, vaccination, nuclear energy, and artificial intelligence. Our work involved three steps. First, we evaluated how well the psychometric paradigm and different embeddings trained on text (*Word2Vec, fastText, GloVe, BERT*) and free associations (*SWOW*) predict the average risk perception associated with each risk. Second, we tested several model ensembles that combine pairs of individual embeddings to investigate the extent to which combinations of text and free association can outperform their single use. This also sheds light on the extent to which the different embeddings encode distinct information. Third, and finally, we compared ensembles of both data sources and the psychometric paradigm, to see whether embeddings can improve the predictive power of the classic paradigm. For all comparisons, we fit the models using regularized regression and evaluated the performance with out-of-sample predictive accuracy using a cross-validation procedure. Figure 2 shows the results of all the three analysis steps. Note that to establish the robustness of our findings and confirm the advantages of relying on the new Basel Risk Norms, we also show analogous results relying on a smaller data set from [15].

Focusing on the comparison of individual models (see Fig. 2A), we observed that the psychometric model is the best-performing individual model, explaining 81.8%, 95% CI: [79.2, 84.3], of the risk perception variance in the Basel Risk Norms. Compared to the results using [15]’s data, this represents a considerable boost, likely attributable to the higher reliability of the Basel Risk Norms and other data quality factors (see Additional file 1). The next best model was the *SWOW* model, an embedding trained on free associations, capturing 74.9%, 95% CI: [72.2, 77.5] of the variance in risk perception. Concerning the text embeddings, we do not see large gains in performance from the recent improvements in training set size and algorithmic architecture. In fact, the earliest text embedding *Word2Vec* remains one of the top-performing text embeddings alongside *GloVe*, respectively capturing 71.8%, 95% CI: [68.4, 75.1], and 71.9%, 95% CI: [68.4, 75.4], of the variance. The models also outperformed the context-aware embedding *BERT*, 67.7%, 95% CI: [63.9, 71.4], suggesting no benefit of the newer model architecture when predicting average risk perception, which in our study consisted mostly of single word risks (e.g., vaccination). All in all, the performance of the free association *SWOW* model is notable, given that it was trained on 100,000 times fewer tokens than the text embeddings, suggesting that free associations represent a rich source of data for predicting risk perception relative to text. The results also show that individual embeddings are close but not, on their own, on par with the prediction performance of the classic psychometric paradigm.

Next, we turned to the comparison of ensembles of embeddings to assess potential performance boosts that may arise from different embeddings possessing nonoverlapping information that can be independently predictive of risk perception ratings (see Fig. 2B). We compared two families of ensemble models: on the one hand, ensemble models composed of pairs of text embeddings, and, on the other, ensemble models based on the combination of text and free-association embeddings. The family of model ensembles involving text embeddings captures 74.1%, 95% CI: [72.4, 75.7], of variance, representing 3.9 percentage points higher performance than single text embeddings. However, ensembles composed of a text and the free-association embeddings performed even better, 79.0%, 95% CI: [77.1, 81.0], leading to a larger improvement in predictive accuracy of 4.2 percentage points over and beyond the free-association embedding—the best individual embedding—and 8.8% over and beyond the text embeddings. Crucially, the best-performing combination of text and free association—*GloVe & SWOW*—scored on par with the classic approach, showing overlapping CI in predictive performance, 79.4%, 95% CI: [77.0, 81.9], with the psychometric model. All in all, these results highlight that an ensemble of text and free-association embeddings can contribute to the prediction of risk perception and rival the performance of the psychometric paradigm.

Finally, we evaluated whether ensembles of models that include the psychometric model can outperform the psychometric model by itself, which would suggest that novel embeddings encode information relevant to risk perception that is not captured by the psychometric model alone (see Fig. 2C versus A). Indeed, we observed that adding embeddings to the psychometric model improves predictive accuracy, with the best addition being that of the free-association embedding *SWOW*, explaining an additional 9.2 percentage points of risk perception variance beyond the psychometric model alone, 91.0%, 95% CI: [89.7, 92.2]. Similarly, a combination of psychometric model with the best ensemble involving text and free-association embeddings accounted for 90.6%, 95% CI: [89.5, 91.8], showing an additional 8.9 percentage points of risk perception variance beyond the psychometric model alone. These results suggest that the psychometric approach can be improved upon by considering additional embeddings.

All in all, these results show that novel embeddings, in particular an ensemble model combining both text and free associations, rival the performance of the psychometric model. They also show that novel embeddings encode information not captured by the psychometric model that can provide a better prediction of risk perception. This provides a basis to investigate the contents of embeddings to potentially uncover novel aspects of risk perception, which we investigate further in the next section.

### Capturing unaccounted dimensions of risk perception

The results of the previous section show that novel embeddings can cover aspects of risk perception not fully captured by the psychometric model. To shed light on these aspects, we carry out an interpretability analysis that involves relating risk perception data to a number of psychological dimensions as captured through word norms; that is, a collation of data sets concerning human-rated (e.g., valence) and other (e.g., frequency) properties of words [e.g., 28]. Our collation of norms can be thought to capture three broad psychological dimensions; namely, affect (valence, dominance, arousal, fear, anger, sadness, disgust, joy, trust, surprise, anticipation), frequency (age of acquisition, familiarity, frequency), and concreteness (concreteness, imageability). Equipped with these data, we conducted a series of analyses aimed at assessing the association of the three groups of norms to risk perception.

Our interpretability analysis consists of three steps. First, we regress the individual norms on the risk ratings to establish a baseline association between each norm and risk perception variance. This baseline can be thought to reflect the importance or ability of each norm in predicting risk perception. Overall, the norms share 64.5% of the variance with risk perception, establishing their usefulness for revealing aspects of risk perception captured by different predictive models. Second, we predict risk perception using the psychometric model and correlate the residuals of the model and the individual norms. The strength of the correlations between the norms and the residuals of the psychometric model can be thought of as systematic variance that is not captured by the psychometric model but can be captured by word norms. Crucially, identifying differences between norm groups (affect, frequency, concreteness) can help find interpretable signals related to this “missing” variance. Third, we predict risk perception using an ensemble of the psychometric model with the best-performing text and free association ensemble, *Psychometric & GloVe & SWOW*, and, again, regress the individual norms on the residuals of the latter model. We then compare the pattern of correlations at baseline (risk variance) relative to those concerning the residuals of the first model, psychometric, and the second model, *Psychometric & GloVe & SWOW*. The rationale for this comparison is that a difference (i.e., drop) between the baseline correlations and those concerning the psychometric residuals will show the extent to which the psychometric model can account for each of the norms. Similarly, a difference in the correlation observed between the correlations with the psychometric model and the *Psychometric & GloVe & SWOW* residuals indicates how much the ensemble model can account for the norms over and beyond the psychometric model.

**Figure 3:**
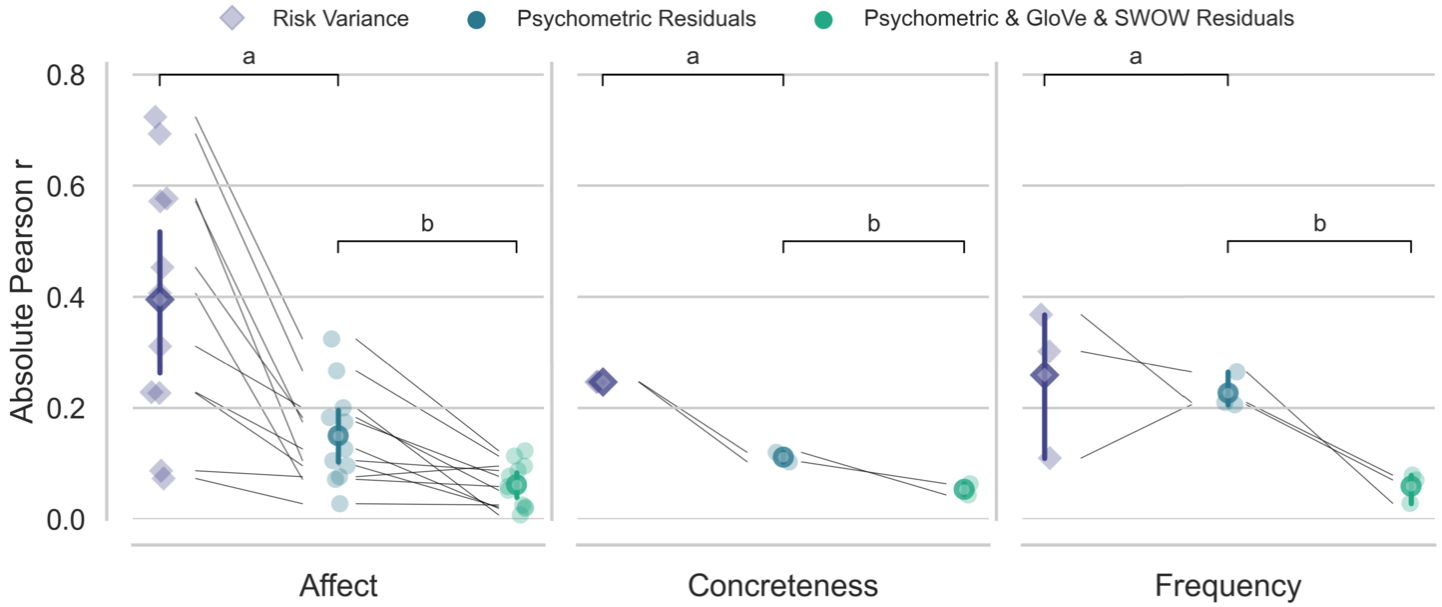
Interpretability analyses. Absolute Pearson correlations of risk ratings, psychometric residuals, and *Psychometric & GloVe & SWOW* residuals with each norm. Norms are grouped into Affect, Concreteness, and Frequency related categories. Foreground points reflect mean correlations for each norm category whereas background points reflect individual correlations with each norm. Connecting lines illustrate the fact that these points are paired. Error bars are 95% confidence intervals

Figure 3 shows the results in terms of absolute Pearson correlations. Considering the baseline correlations, labeled *Risk Variance*, it can be seen that affect-related dimensions are most important to risk perception, $r=0.40$, relative to concreteness, $r=0.25$, and frequency, $r=0.26$. Most importantly, as can be seen by attending to the comparison brackets *(*a), predicting risk perception using the psychometric model leads to substantial drops in the correlations of at least two norm groups, specifically, affect norms—with an average drop, $\Delta =0.25$, in correlation—and concreteness-related norms—with an average drop of $\Delta =0.14$—but a negligible drop regarding frequency ($\Delta =0.03$). Notably, many individual affect, concreteness, and frequency norms still showed sizable correlations when considering the psychometric residuals. These results suggest that although powerful, the psychometric model is unable to fully account for the signals of affect, concreteness, and frequency that are systematically related to risk perception.

Can the embedding ensemble account for norm signals unaccounted for by the psychometric approach? To a large extent, this is the case. As can be seen by attending to the comparison brackets *(*b), on average, the residuals of the *Psychometric & GloVe & SWOW* ensemble show descriptively smaller signals for all three norm groups than psychometric residuals, with substantial drops in correlation for affect ($\Delta =0.09$), a small drop ($\Delta =0.06$) for concreteness, and a somewhat larger drop for frequency ($\Delta =0.17$). These results highlight that the novel ensemble involving text and free-association embeddings capture much of the unaccounted norm signals, particularly those related to affect and frequency.

Finally, it is worth noting that even after accounting for the psychometric model and the embedding ensemble, signals for some but not other norms remained larger than zero. This was the case, for instance, for some affect-related norms but also frequency norms. These remaining signals point to missing aspects that may help to further improve the prediction of risk perception.

All in all, the results of our interpretability analysis show that the embeddings help capture key aspects related to affect and frequency that the psychometric model considered here did not fully capture. They also provide us with an improved understanding of the abstract representations involved in the ensemble model, suggesting that there is a broad set of aspects at play, related to affect, frequency, and concreteness. Issues of interpretability are pivotal when considering applications in the real world that require understanding and justification of model performance [e.g., 29–31].

### Exploring the applicability of embeddings for real-world prediction

The strong performance and large vocabularies of embeddings open up opportunities to apply these models beyond the ratings in our new risk data set to predict the anticipated risk perception in real-world situations. To demonstrate this, we analyze news headlines [32]. News headlines are a real-world example of text documents that are useful to predict the associated risk perception. Such predictions could, for instance, be used to better understand social trends or to improve risk communication. However, predicting the risk perception for a news headline requires that the headline contains words that are also in the given risk model’s vocabulary. This highlights an important evaluative criterion of a risk model that goes beyond prediction accuracy. Namely, the extent to which the model can be applied in linguistically diverse environments, as determined by the size and relevance of the model’s vocabulary.

**Figure 4:**
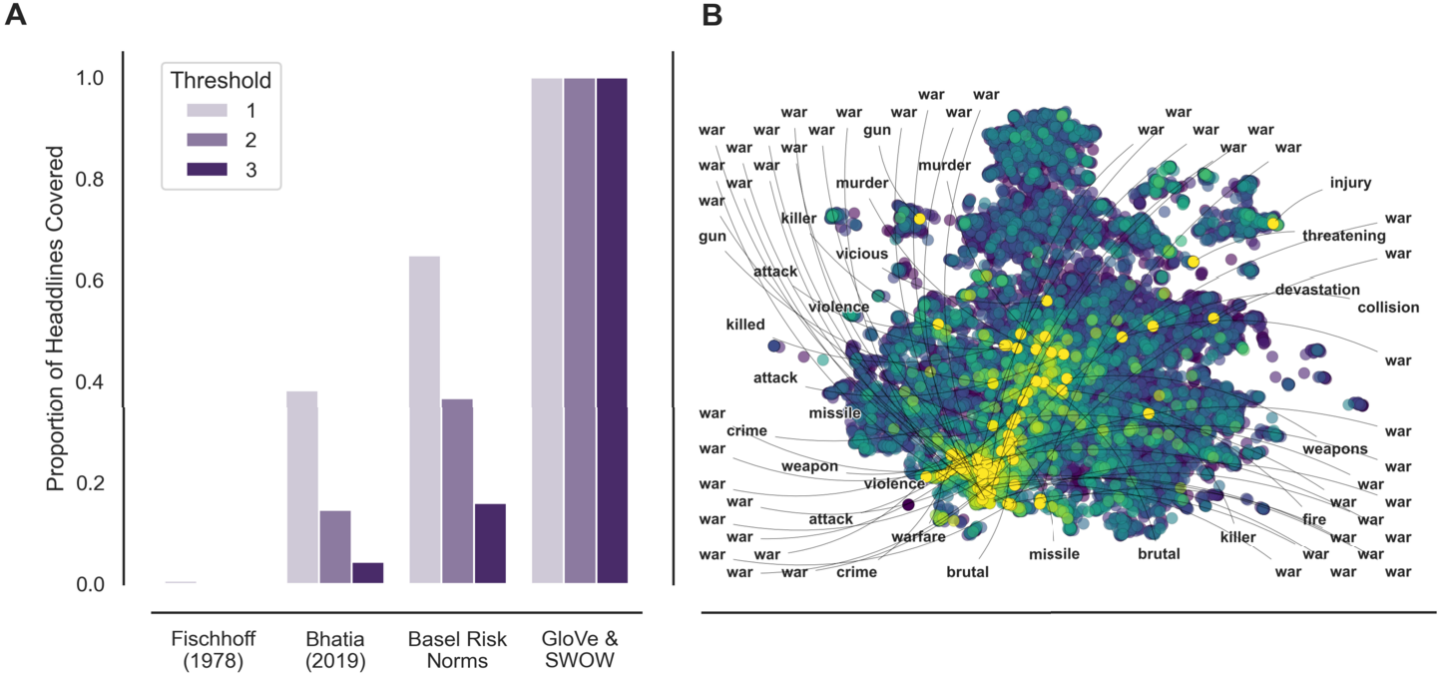
Coverage of risk vocabularies in news headlines. **A.** Proportion of news headlines ($n=15{,}031$) covered, where coverage is defined at different thresholds (at least one word, two words, or three words can be predicted by the model). Vocabularies include those of [7] (30 risks), [15] (428 risks), Basel Risk Norms (1004 risks), and *GloVe & SWOW* (11,895 words). **B.** UMAP visualization of each headline, with headlines colored according to the mean riskiness of the words contained according to the text and free association ensemble model (*GloVe & SWOW*). Headlines with mean riskiness greater than an arbitrary threshold are labeled with the riskiest noun or verb in the headline (75 headlines). One label was removed due to its being a trigger word

We carry out the following analysis to compare the real-world applicability of the different risk models used above. First, we computed the proportion of headlines that contained words included in the vocabulary of the best-performing embedding ensemble (*GloVe & SWOW*) and two previous psychometric risk norm sets. Specifically, we considered the vocabularies included in [7, 15], and the new Basel Risk Norm data set. We carried out this analysis for three criteria—covering at least one, two, or three words—reflecting different levels of information about the contents of the headlines. The results are shown in Fig. 4A. For the liberal criterion of at least one word, we observed coverages of less than 1% [7], 38% [15], 65% (Basel Risk Norms), for the three psychometric vocabularies, and 100% for the embedding ensemble (*Word2Vec & SWOW*). This means that although the larger vocabulary of the Basel Risk Norms increases the coverage twofold over the largest previously available vocabulary [15], it nevertheless fails to cover one-third of the headlines. Crucially, coverage of the psychometric vocabularies dropped dramatically when requiring that they contain two or three words of the headlines, such that the largest psychometric vocabulary, the Basel Risk Norms, merely covered about 16% of headlines at the three-word threshold. By contrast, the embedding ensemble—which contains vocabulary that is an order of magnitude larger than that of the Basel Risk Norms—remains at 100%, highlighting the applicability of embeddings for predicting risk perception in real-world contexts.

In addition to coverage, we provide a proof of concept for predicting risk perception by analyzing the headlines that, according to the *GloVe & SWOW* embedding, are high in risk perception. To this end, we first projected the 15,031 headlines into a two-dimensional semantic space. Specifically, we applied the “Uniform Manifold Approximation and Projection” (UMAP) algorithm, a nonlinear dimensionality reduction technique [33], to the headline-token frequency matrix transformed using the term-frequency-inverse-document-frequency normalization. We then predicted the risk perception for each headline using the best-performing embedding ensemble—*GloVe & SWOW*—by averaging the estimated riskiness of the words included in the headline and scaling the mean predictions across headlines to lie between −100 and 100. Figure 4B shows headlines as points with color representing the predicted riskiness and the tokens with the highest predicted perceived riskiness for each of the top 75 most risky headlines. As one would expect, the predicted perceived riskiness clusters within the semantic space, reflecting that those headlines semantically similar to others with perceived riskiness also are high in perceived riskiness. Considering the high-risk tokens, these headlines form distinct groups of high-risk topics, such as war, murder, attack, or crime, justifying the numerical riskiness assigned to the headline. As a whole, these results underscore the potential of using embeddings to generalize from risk-rating data to the prediction of riskiness for larger, socially relevant text units.

## Discussion

Our investigation asked whether novel embeddings trained on text and free associations can help predict risk perception over and beyond the classic psychometric paradigm. To answer this question, we generated the largest and most reliable available risk norms involving ratings of 1004 risks, from vaccination to artificial intelligence. Our results led to four key findings. First, although the psychometric paradigm outperformed all individual embeddings, an ensemble of embeddings trained from text and free association showed comparable performance. Second, adding the novel embeddings to the psychometric paradigm substantially increased the performance of the psychometric model, reaching an extremely high predictive accuracy of over 90% of variance explained. Third, as revealed by our interpretability analysis, the novel embeddings provide additional predictive validity by accounting for affective and frequency-related aspects of risk perception that the psychometric paradigm did not fully capture. Fourth, and finally, the analysis of a set of over 15,000 news headlines demonstrated that the larger vocabulary of embeddings by far extends the coverage of the psychometric paradigm and can be leveraged to generalize risk prediction to words and texts in real-world settings. These results highlight the utility of novel embeddings, in particular a combination of those derived from text and free associations, to improve our understanding and prediction of risk perception. They also demonstrate the importance of behavioral data for both training and interpreting language embeddings used in data science more broadly.

Our findings have important implications for the psychological study of risk perception. For decades, researchers working within the psychometric paradigm have relied on their ability to devise a comprehensive set of survey questions to understand and predict risk perception [7, 9]. We show that novel embeddings that have recently become available now present a promising alternative to the classic paradigm, expanding past work [15], by showing that novel embeddings not only rival the predictive performance of the classic psychometric model but also capture unique aspects of the phenomenon that the classic model used in many past investigations fails to capture. We should note that our results do not represent a rejection of the insights derived from the psychometric model. The large overlap in predictive ability between the psychometric and the novel embeddings approaches suggests that the features derived manually from past work with the psychometric paradigm were well tuned to the task of understanding and predicting risk perception. Nevertheless, our results show that novel embeddings demonstrate similar performance and can even be used to extend the psychometric approach to include aspects associated with affect or familiarity of risks. These insights can be directly included in future empirical work with the psychometric paradigm by extending the dimensions captured by psychometric surveys, and the interpretability analysis used to obtain these insights helps establish a new approach for data scientists to understand the (differing) outputs of language embeddings.

Another important implication concerns the ability to predict risk perception beyond a small vocabulary of risk sources that has been typically considered in the risk perception literature. One powerful feature of embeddings is that they provide quantitative representations of tens of thousands of words. Using the insights from our analysis, it is possible to generalize the prediction of risk perception to new words with high accuracy. We have shown that this can be exploited to predict risk perception associated with larger units of text, such as news headlines. We think that down the line, such applications will prove useful in evaluating and designing communication concerning current and novel types of risks. For example, such approaches could be used to track risk perception driven by polarization in news content over time [e.g., 34] or tracking the effects of specific events, such as natural or technological catastrophes, on the public’s risk perception from various text sources, such as social media [e.g., 35, 36].

A final implication concerns the data source of embeddings. To produce accurate word embeddings, language models are typically trained on gigabytes of digitized text; however, our analysis has shown this approach can be improved upon by relying on appropriate data. Specifically, we found that embeddings constructed using free association data can outperform those constructed from text, although they were trained on 100,000 times fewer data. This suggests that behavioral data, such as free associations, may provide a rich data source for predicting not only risk perception, but also other psychological and behavioral outcomes that may be of interest to data scientists more generally. Future work may want to consider comparing and extending existing text-based models with additional behavioral sources to provide predictions of human judgments and behavior [e.g., 13, 21, 37, 38].

There are some limitations of our work worth highlighting. First, our results are based on aggregate data. As a result, we did not distinguish between demographic groups, essentially averaging over males and females, or younger and older individuals. Yet, there are important individual and group differences in people’s understanding of risk [39]. Future work should explore the role of individual and group differences in the predictive ability of language embeddings for risk perception applications. Some promising applications include the use of embeddings derived from behavioral data that can be obtained for specific demographic groups [21], or the application of large language models that allow demographic steering through prompting to investigate the models’ abilities to capture such demographic variation. Second, our analysis was only carried out in English, with first language English speakers, potentially ignoring cultural–linguistic differences in risk perception, which are also known to vary considerably [e.g., 40]. One possible avenue for future work could involve examining the predictive value of novel embeddings across languages and cultures. Third, the Basel Risk Norms consist of single words or bigrams and are imperfectly suited to capture contextual aspects of a given risk. Future investigations should explore risk perception in more naturalistic linguistic contexts, for example, by evaluating human judgments of larger units of text in different contexts (e.g., print media, social media). In such settings, we predict that the new class of context-aware embeddings (i.e., transformers) will, ultimately, be of greater use.

In conclusion, we assessed whether the prediction of risk perception can be improved by novel approaches relying on language embeddings. Our results demonstrate how this novel approach can successfully predict aggregate risk ratings, elucidate its psychological underpinnings, and track risk perception elicited by news headlines. All in all, our results establish the ensemble of text and free-association embeddings as a powerful new tool to deliver the longstanding promise of tracking risk perception in real-world settings.

## Methods

### Data

#### Basel risk norms

The first step in developing the Basel Risk Norms consisted in generating a list of risks that can plausibly be understood as a risk, irrespective of whether the perceived riskiness would be high or low. To this end, we developed an algorithm that consisted of the following steps. First, we identified a large list ($N = 10{,}351$) of nouns and verbs included in all embedding vocabularies. Second, human voters rated each word on whether or not it can be interpreted as a risk. Third, using embeddings (*fastText*, *GloVe*, and *SWOW*) we evaluated the semantic similarity of words to the risks studied by [15] and [8] and rated the words as risks when the similarity exceeded a threshold that was selected to match the rate of human positive responses. Fourth, we included a risk in a preliminary list when it received either two human votes, or one human vote and at least two out of three machine votes. Finally, we filtered the preliminary list by excluding words that were of very low frequency (e.g., “barracuda”), shared lemmas with other words in the list (e.g., “ashes” and “ash”), and were sensitive (six words). Ultimately, this algorithm led to a list of 1004 risks.

We collected two participant samples via Prolific Academic to provide ratings for the risk item and the nine psychometric items (see Table 1), respectively. The risk sample consisted of 1506 participants, with an average age of 40.4 years and 47% female gender. Each participant evaluated a pseudo-random 100 risks on a scale from –100 to 100, consistent with [15]. The psychometric sample consisted of 2360 participants, with an average age of 39.7 years and 49% female gender. Each participant rated 20 risks on a scale from 1 to 7 concerning each psychometric item. The items were presented on separate pages and in two orders. The reverse order performed 6.53% better than the original order on average (see Additional file 1). The sample sizes were selected to achieve reliabilities of ($\rho _{\mathrm{risk}}=0.995$) and ($\rho _{\mathrm{psych}}=0.97$) for the risk and psychometric ratings, respectively, which closely matched the recorded reliabilities of .995 and .95. Participants were compensated with a median rate of 7.37 GBP per hour.

We took several steps to ensure high data quality. First, we only selected participants with a minimum approval rating of 95%. Second, we included an initial check, whereby participants could commit to providing thoughtful answers to the questions in the survey [41]. Third, we included three attention checks placed at different points in the assessment and, in accordance with Prolific’s policy, excluded participants who failed more than one attention check. Fourth, we split the assessment into several sessions; specifically, we distributed the assessment across multiple occasions over the course of two consecutive weeks (Monday to Sunday), with sessions at 10 am and 4 pm GMT each day. Data collection was completed on October 10, 2022, preceding the public launch of ChatGPT.

#### Data from Bhatia (2019)

The data by [15] includes three data sets of psychometric and risk ratings. The first two sets contain 125 technologies and 125 activities based on [8], whereas the third contains 200 risks generated by participants in a free-association task. Of these 200 risks, 21 overlapped with the first two sets, resulting in 429 unique risks in total. Taking the intersection with the different embedding vocabularies reduced this set to a final set of 306 unique risks for the analysis. All three studies were collected using crowd samples from Prolific.

In order to collectively analyze the risk and psychometric ratings from these three data sets, we joined them and calculated the risk-wise means for each psychometric item and the risk item. This differs from the strategy used in [15], where a psychometric model is obtained from each data set separately and evaluated only on the risks in that data set. Nevertheless, we find our approach of aggregating all three data sets to be on par with the best-performing psychometric model reported in [15], suggesting that our aggregation did not hinder model performance.

#### News headlines

The data contain headlines from the British Broadcasting Corporation (BBC), including a title, publication date, GUID, link, and description. It was scraped from a self-updating RSS feed via a kernel hosted on kaggle.com [32]. We used a version of the data available on March 23, 2023 (the data set is updated on a daily basis), which contains 15,031 headlines from May 9, 2017 until March 23, 2023.

### Embeddings

#### Text embeddings

Our analysis of text embeddings draws on the following pretrained models. First, *Word2Vec* is a neural network-based embedding that employs the continuous-bag-of-words (CBOW) model architecture, whereby the model is trained by predicting words from other close-by words across a large amount of text [42]. The model used in our analysis was trained on the Google News data set, including roughly 100 billion words. Second, *fastText* also uses a CBOW architecture, but improves upon *Word2Vec*, for instance, by implementing a position-dependent context weighting [17]. *fastText* was trained on the Common Crawl, a corpus of web pages that is more diverse and with 600B tokens considerably larger than the corpus used to train *Word2Vec*. Third, *GloVe* implements a matrix factorization approach that seeks to combine the advantages of “local context window” approaches such as *Word2Vec* and *fastText* and “global matrix factorization” methods such as singular value decomposition [16]. *GloVe* was trained on a slightly larger version of the Common Crawl than *fastText* comprising 804B. Finally, *BERT* implements a new class of the transformer neural network architecture. There are several ways to extract embeddings from *BERT*. We extract risk-level embeddings by feeding in the entire risk item; that is, “How risky or safe is the following?: X”, with X being each risk. Although this is a relatively brief input for a transformer, we hypothesized that the additional context provided by the risk item could lead *BERT* to give greater attention-weight to hidden dimensions relating to risk [14, 43]. We reasoned that this might provide *BERT* with an advantage over the other embedding approaches because it could increase the signal of risk-relevant information in the extracted features. BERT was trained on the BookCorpus containing 11,000 unpublished books and English Wikipedia [18].

#### Free-association embedding

The free-association embedding used in our analysis was trained by us. As the data source, we used publicly available data from the citizen-science Small World of Words (SWOW) study in English [22], which contains associations to 12,282 cues from over 90,000 participants. Using these data, we employed the following three-step procedure. First, we transformed the data into a cue–response matrix **M** with 12,282 rows reflecting the cues and 32,312 responses with a response frequency larger than 5. Second, we generated the matrix $\mathbf{M^{\prime}}$ from **M** by computing the positive point-wise mutual information between cues and responses, which is a frequent metric employed in the domain of computational semantics to account for word frequency effects [see, e.g., 44]. Third, we applied truncated singular value decomposition to $\mathbf{M\prime}$ to obtain 300-dimensional embeddings. Specifically, we used **UΣ**, the left-hand vectors times the singular value diagonal matrix, from $\mathbf{M^{\prime}}=\mathbf{U}\boldsymbol{\Sigma}\mathbf{V^{*}}$.

#### Ensemble models

Ensembles were generated by concatenating the individual embeddings. For instance, the ensemble of Word2Vec (300D) and SWOW (300D) results in a 600-dimensional embedding ensemble consisting of all dimensions from either embedding. As explained below (section “Prediction of risk perception”), in contrast to common practice, we did not standardize the individual predictors in the regularized regression. Nevertheless, to account for the fact that the embedding dimensions of different models composing each ensemble can systematically differ in their scaling, we performed a groupwise scaling such that the mean standard deviation of the dimensions of each model equaled the mean standard deviation of the psychometric model. Equalizing the mean dimension scaling between models ensured that the average regularisation penalty per dimension was applied uniformly across each model composing the ensemble. Thus, whilst the individual dimensions were not penalized equally (for reasons explained below) the individual embeddings in each ensemble were.

### Prediction of risk perception

We predicted the risk ratings using elastic net regularized regressions. As we evaluated models with between nine (psychometric model) and 609 (embedding–psychometric ensemble) predictors, regularization was necessary to avoid overfitting. We used cross-validation to identify the best mix of penalty types (L1 or L2) or penalty magnitude *α*. Specifically, we evaluated a grid defined by 11 even steps in the interval of $l1\_\mathrm{ratio} \in [0, 1]$ and eight exponential steps in the interval of $\alpha \in [10^{-5}, 100]$. In addition to elastic net regression, we evaluated gradient boosting as a nonlinear predictive algorithm. Because the gradient boosting did not perform better than elastic net for all but one model (*Psychometric* performed 2.5 percentage points better with gradient boosting), we have placed the gradient boosting results in the Additional file 1.

Model performance was assessed with nested 10-fold cross-validation repeated 10 times with random shuffles of the data on each repeat. This nested strategy is native to the Scikit-Learn API, which we used for our analysis, and is recommended by [45] to prevent data leakage. The strategy works by fitting the model (hyper)parameters in an inner loop composed of training and validation sets. Generalization performance—in our case measured using the coefficient of determination ($R^{2}$)—is then computed on a held-out test set in the outer loop. We did not standardize the predictors in elastic net regression. The reason for this was because it had small and inconsistent impacts on the performance of the text embeddings whereas it consistently negatively affected the performance of the free-association embedding (see Additional file 1). The negative effect on the performance of the free-association embedding is likely due to the use of singular value decomposition, which distributes variance highly unevenly across the resulting embedding.

### Preregistration

The data collection and planned model comparisons were preregistered at [46]. Compared to the preregistered analyses, we included additional embeddings. Specifically, we included BERT, in order to provide a reference to newer generation language models, and additional ensemble models. These additional ensembles include all pairwise text-embedding combinations and all psychometric, text, and free-association ensembles beyond *Psychometric & GloVe & SWOW*. We include these to provide a more comprehensive overview of how the different models complement each other.

### Supplementary Information

Below is the link to the electronic supplementary material.

(PDF 2.3 MB)

## Data Availability

With the exceptions of the full versions of SWOW and any participant-identifying information, all data used for this study, code, and supplementary materials are available in the ‘Semantic Accounts of Risk Perception’ repository, https://osf.io/gu9df/ [47], in accordance with the FAIR principles. A version of SWOW containing all cues is available from https://smallworldofwords.org/en/project/research (SWOW-EN2018) [22], but cannot be redistributed for licensing reasons.

## Abbreviations

*GloVe*: Global Vectors
*BERT*: Bidirectional Encoder Representations from Transformers
*SWOW*: , Small World of Words study in English;
UMAP: Uniform Manifold Approximation and Projection
BBC: British Broadcasting Corporation
