# Supplementary material for: Novel embeddings improve the prediction of risk perception
Source: EPJ Data Sci. 2024 May 22;13(1):38. doi: 10.1140/epjds/s13688-024-00478-x (PMC11111540; doi:10.1140/epjds/s13688-024-00478-x)
Supplement: Supplementary file 1 — (PDF 2.3 MB) [file 13688_2024_478_MOESM1_ESM.pdf]

Supplementary materials for 'Novel embeddings improve the prediction of risk perception'

Zak Hussain[1,2*], Rui Mata[1†] and Dirk U. Wulff[2,1]

[1*]Faculty of Psychology, University of Basel, Missionsstasse 62a, Basel, 4055, Switzerland.
[2]Center for Adaptive Rationality, Max Planck Institute for Human Development, Lentzeallee 94, Berlin, 14195, Germany.

*Corresponding author(s). E-mail(s): z.hussain@unibas.ch;
Contributing authors: rui.mata@unibas.ch; wulff@mpib-berlin.mpg.de;
†These authors contributed equally to this work.

1 Data collection

We followed common procedures used in the risk perception literature to obtain data for the psychometric paradigm [e.g., 1, 2]. The pre-registration for the study is available at https://osf.io/6m7xr. In what follows, we investigate the sensitivity of our results to various factors surrounding data collection. We focus on two main factors: the impact of psychometric item ordering—which could affect both predictive accuracy and inter-item correlations—and the impact of training set size (with a focus on predictive accuracy).

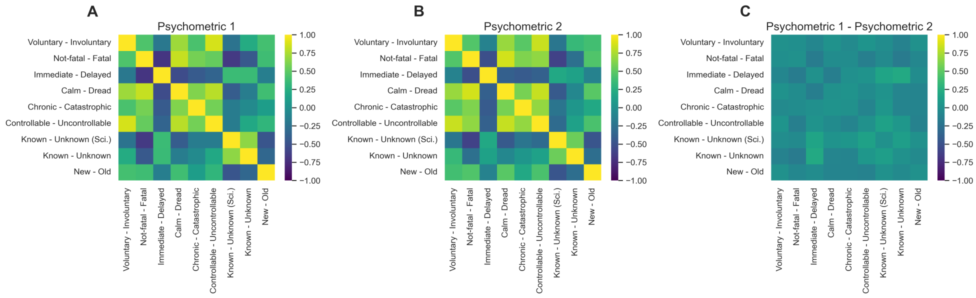

Fig. 1 Investigating the impact of psychometric item ordering on inter-item correlations. **A.** Order 1 inter-item correlations. **B.** Order 2 inter-item correlations. **C.** Order 1 minus order 2 inter-item correlations.

1.1 Impact of psychometric item ordering

In our survey, half of the participants received the psychometric items in the order presented below (order 1) for each risk and the other half received them in the reverse order (order 2). The main reason for doing this was to investigate whether ordering actually impacts participant responses, which, to our knowledge, has not been done before, and could affect data quality.

1. **Voluntary–Involuntary**—Are individuals exposed to this risk voluntarily or involuntarily?
2. **Immediate–Delayed**—Is death from this risk immediate or delayed?
3. **Known-Unknown**—Is this risk known or unknown to the individuals exposed to this risk?
4. **Known–Unknown (Sci.)**—Is this risk known or unknown to science?
5. **Controllable–Uncontrollable**—Is this risk controllable or uncontrollable for the individual exposed to the risk?
6. **New–Old**—Is this risk new or old?
7. **Chronic–Catastrophic**—Is this a risk that kills one person at a time (chronic) or a risk that kills large numbers of people at once (catastrophic)?

8. **Calm–Dread**—Is this a risk that individuals can reason about calmly or is it one that they have great dread for?
9. **Not-fatal–Fatal**—How fatal are the consequences of this risk?

To evaluate potential differences between the two orderings, we carried out several analyses. First, we focus on the psychometric ratings alone. To investigate whether psychometric ordering had a statistically significant impact on responses, we take the individual ratings for each risk source and psychometric item, split them into two groups (order 1 and order 2), and run an independent-samples t-test on each pair of groups. This amounted to 9,036 t-tests (1,004 risks times 9 psychometric items), of which 11.6% of the groups significantly differed for $\alpha = .05$. This is twice the number of type I errors expected, suggesting a small influence of ordering on average responses. Four out of the nine items (*Immediate–Delayed*, *Voluntary–Involuntary*, *Calm–Dread*, and *Known–Unknown*) account for almost 60% of the significant differences. However, overall, the difference in the average responses was small (average Cohen's $d = .09$). Furthermore, the average ratings in the nine psychometric items showed very high Pearson correlations of, on average, 0.88.

We further evaluated the robustness of the inter-item correlation between the two orderings because this has implications for the sensitivity of principal component analyses (PCA) often performed within the psychometric paradigm [cf. 1]. Figure 1 shows the correlations across risks between psychometric item ratings for both orderings. We observed very similar patterns of correlations but also small differences ranging from $\delta < .001$ (*Immediate–Delayed* and *Chronic–Catastrophic*) to $\delta = .21$ (*Immediate–Delayed* and *Known–Unknown*), with an overall average absolute difference of $\delta = .08$.

Finally, we evaluated potential differences in the accuracy of predicting risk perception (See Figure 2). We observed that *Psychometric 2* achieved a 6.4 percentage

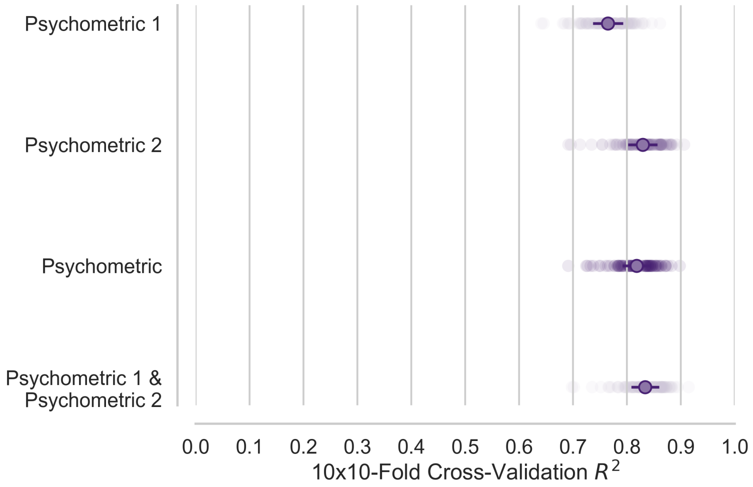

Fig. 2 Investigating the impact of psychometric item ordering on performance. *Psychometric 1* is obtained from participants that received the following order 1 (as listed in text). *Psychometric 2* participants received the reverse order. *Psychometric* is an aggregate of orders 1 and 2 (as used in the main analysis), and *Psychometric 1 & Psychometric 2* is the concatenation of both orderings. Error bars are adjusted 95% confidence intervals [3].

points higher accuracy than *Psychometric 1* and a 1.1 percentage points higher accuracy than the aggregate psychometric model using both orders. This means that the reversed order is better at capturing risk perception than the original order. This may have contributed to the higher performance of the psychometric model in the Basel Risk Norms compared to the data of [2] because the latter relied only on the first ordering. The notable differences in predictive accuracy between the two orders have two noteworthy implications. First, other orderings of psychometric items could result in even larger predictive accuracy for the psychometric model. Second, the two orderings may capture distinct aspects of risk perception, suggesting that they might best be used in tandem rather than aggregated. To test the latter, we evaluated the concatenation of both orderings, *Psychometric 1 & Psychometric 2*, as a predictive model. We observed that the concatenated model outperformed the aggregate model by 1.6 percentage points, which is a small but significant effect ($t = 4.00, p < .001$).

Overall, our evaluation of orderings revealed some differences in average ratings, inter-item correlations, and predictive accuracy. However, the differences between orderings were overall small in magnitude. Furthermore, although the slightly higher accuracy of the concatenated model compared to the aggregate model may justify using the concatenated from the perspective of predictive accuracy, this choice would disadvantage our analysis in other ways. Specifically, it would limit interpretability, given that we possess no information on how the item ordering affects the content of the responses to the psychometric items, and comparability to previous work including, in particular, the study by [2]. We believe that the small gains in accuracy do not outweigh these costs, and so chose to use the aggregate model.

1.2 The impact of training set size on predictive accuracy

In planning the data collection of the Basel Risk Norms, we investigated the potential of increasing predictive accuracy by increasing the training set size. We trained different models on different portions of the data of [2] and recorded the accuracy of predicting risk perception (see Figure 3; green lines). The analysis showed significant potential for higher accuracy, with accuracy values increasing systematically with larger training set sizes. The increasing accuracy is likely due to a decreasing role of model overfitting. This potential for increased accuracy suggested by the reanalysis of the data of [2] was largely realized by the larger Basel Risk Norms. Figure 3 also shows the accuracies of the different models for the Basel Risk Norms, which demonstrate clear performance increases for the larger training set sizes.

Three additional results concerning the relationship between training set size and predictive accuracy in the Basel Risk Norms are worth noting. First, the accuracies appear to taper off for larger training set sizes. One important implication of this is that comparisons between the low-dimensional psychometric model and the high-dimensional embeddings models are fairer using the larger Basel Risk Norms. Second,

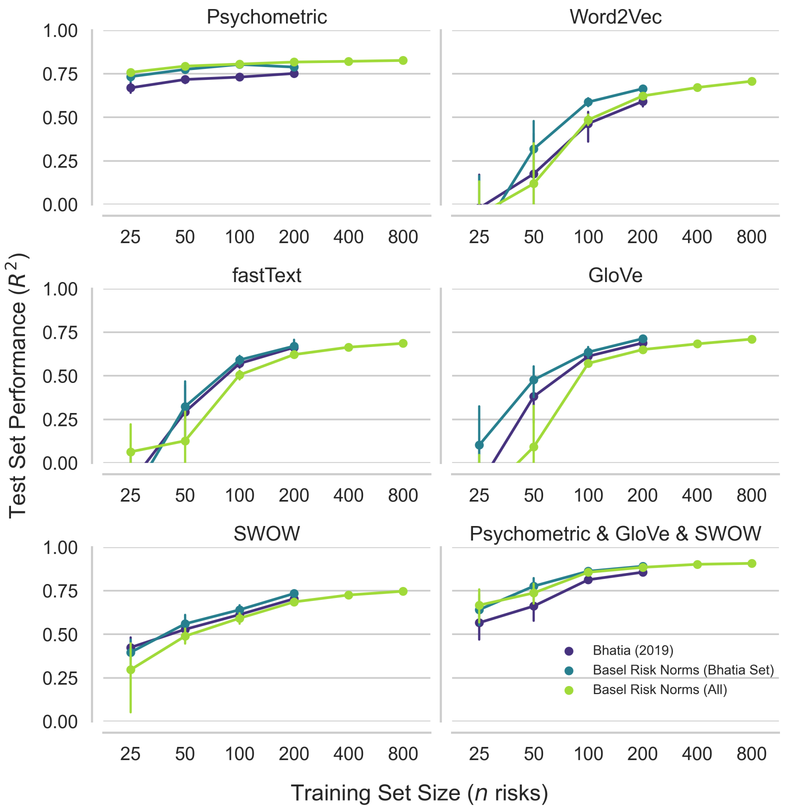

Fig. 3 Evaluating how test performance varies with training set size for 3 data (sub-)sets: (i) Basel Risk Norms (All), which refers to our full data set of 1,004 risks, (ii) Basel Risk Norms (Bhatia Set), referring to our data limited to the same 306 risks as used in [2], and [2] (iii). Test sets are composed of all remaining risks in the data. Train–test splits were sampled randomly (i.e., bootstrapped), with 10 repetitions per model per training set size. Error bars are 95% confidence intervals.

the accuracy of the psychometric model is systematically higher for the Basel Risk Norms compared to the data of [2] for any training set size. This difference likely reflects the substantial increase in reliability due to a larger number of ratings. Third, embedding accuracies for small training sets are worse for the Basel Risk Norms than the data of [2] when considering all risks and better when considering only the risks shared across data sets. These results are consistent with the higher risk rating reliabilities of the Basel Risk Norms but also suggest that the newly introduced risks may result in a larger diversity of risks, making it harder to generalize from train to test set.

Overall, by increasing the size of the risk set, we boosted the performance of all models thus permitting a fairer comparison of model performance due to less model overfitting.

2 Model comparison

In this section, we provide additional information concerning the sensitivity of our model comparison results to various analytic choices. We first justify our decision to focus only on the results of a linear regression algorithm (elastic net) in the main paper, instead of more flexible nonlinear methods such as the popular gradient boosting. We next motivate our decision to use a groupwise scaling technique during pre-processing, instead of more traditional approaches to scaling preceding regularized regression such as standardization. Finally, we provide a comprehensive statistical analysis of the differences between all pairwise model combinations for completeness.

2.1 Elastic net versus gradient boosting

In addition to elastic net regression, we evaluated the predictive accuracies of the different models using Scikit-Learn's gradient boosting regressor [4]. Gradient boosting is a popular nonlinear algorithm that builds an additive model out of regression trees in a forward stagewise fashion. In many cases, gradient boosting can outperform linear models, especially when more training samples are available.

We observed that for all but one model gradient boosting was at best equal and, in many cases, clearly worse than the linear model (see Figure 4). The only exception was the low-dimensional psychometric model, which saw a small increase in the predictive accuracy of 2.6 percentage points on the Basel Risk Norm data. Interestingly, we also see the impact of the increased training set size, with the additional risks in our norm set reducing the relative advantage of elastic net over gradient boosting. This indicates

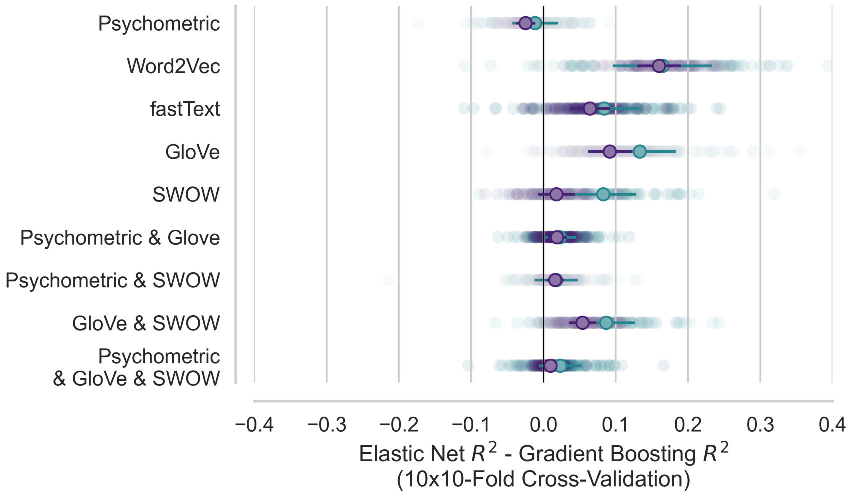

Fig. 4 Pairwise differences between elastic net and gradient boosting using 10x10-fold cross-validation. Cross-validation via [2]'s risk norms (306 risks) are colored cyan and points obtained using the Basel Risk Norms (1004 risks) are colored purple. Error bars are adjusted 95% confidence intervals [3].

that perhaps with a sufficient number of samples, the more flexible gradient boosting model could outperform elastic net.

Overall, regularized linear regression emerged as the superior model, which is consistent with the relatively low ratio of data points to features.

2.2 Evaluating embedding scaling approaches

When relying on regularization techniques, such as elastic net regularization, it is common practice to standardize the predictors to even out their contribution to the regularization penalty. However, we based our analysis on unstandardized embeddings. We did this to allow for a fair comparison between the free-association and text embeddings. The free associations embedding ($SWOW$) was trained using singular value decomposition, which by design allocates variance very unevenly across the embedding dimensions. Standardizing $SWOW$ would thus imply removing an important prior on the importance of embedding dimension, which can result in reduced predictive accuracy. To quantify the potential negative effect of standardization on $SWOW$ and a

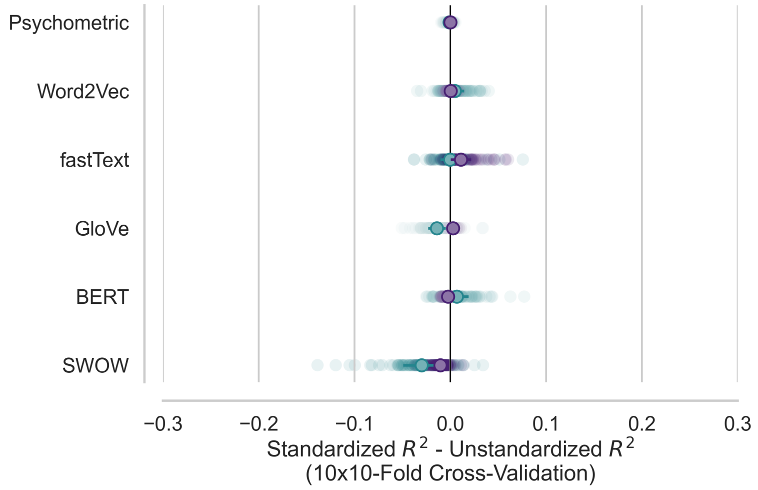

Fig. 5 Pairwise differences between standardized and unstandardized models using 10x10-fold cross-validation and elastic net regression. Cross-validation via [2]'s risk norms (306 risks) are colored cyan and points obtained using the Basel Risk Norms (1004 risks) are colored purple. Error bars are adjusted 95% confidence intervals [3].

potentially positive effect for the other embedding models, we explicitly compared the predictive accuracy for every model with standardized and unstandardized dimensions for both risk norm sets (Bhatia, 2019, and Basel Risk Norms).

As can be seen in Figure 5, standardizing did indeed negatively impact the *SWOW*) for both norm sets (Bhatia, 2019: $t = -3.09, p = .003$, Basel Risk Norms: $t = -3.68, p < .001$). In terms of the text embeddings, the effect of standardizing was mixed, with a negative effect for *GloVe* on [2]'s data ($t = -3.04, p = .003$), and smaller positive effects on the Basel Risk Norms for *GloVe* ($t = 2.26, p = .027$) and *fastText* ($t = 2.05, p = .043$). *Psychometric* was not significantly affected. In light of these findings, we chose not to standardize the models in our analysis.

2.3 Statistical tests

The comparison of models was carried using the procedure described in [3] (see also, [5]). It involves calculating the differences in model performance across the same 100 (10x10) train-test splits for each pair of models and testing the null hypothesis that

the mean difference equals zero using an adjusted paired t-test that accounts for the dependence between train-test splits.

To give an overview of all possible model comparisons, Figure 6 shows the differences in R-squared predictive accuracy for all pairs of individual and ensemble models (y-axis models minus x-axis models) with nonsignificant differences displayed as white.

Several important insights emerge from the patterns of results. First, the patterns of results are highly similar between the data of [2] and the Basel Risk Norms, with one exception being the large number of significant results for Basel Risk Norms due to the higher reliability and larger data set size. Second, ensembles containing the psychometric model outperform ensembles without the psychometric model, as indicated by the strong bright rectangle in the bottom left corners. Third, there is only one model not significantly different from the psychometric model—*GloVe & SWOW*—attesting to the strong performance of *SWOW* in capturing important aspects of risk perception.

3 Word norms

Our interpretability analysis identified unaccounted dimensions of risk by relying on a set of word norms. For this purpose, we selected a set of norms from [6] that we hypothesized to be related to risk perception. Table 1 provides an overview of these norms and lists the individual sources. As reported in the main text, these norms are able to predict 64.3% of risk perception variance (with 32% of the norm data imputed using *Word2Vec* to deal with missing norm data on certain risks), establishing their usefulness for revealing the key aspects of risk perception.

References

[1] Fischhoff B, Slovic P, Lichtenstein S, et al (1978) How safe is safe enough? a psychometric study of attitudes towards technological risks and benefits. Policy Sciences 9(2):127–152. URL https://doi.org/10.1007/bf00143739

[2] Bhatia S (2019) Predicting risk perception: New insights from data science. Management Science 65(8):3800–3823. URL https://doi.org/10.1287/mnsc.2018.3121

[3] Bouckaert RR, Frank E (2004) Evaluating the replicability of significance tests for comparing learning algorithms. In: Pacific-Asia Conference on Knowledge Discovery and Data Mining, Springer, pp 3–12, URL https://doi.org/10.1007/978-3-540-24775-3_3

[4] Pedregosa F, Varoquaux G, Gramfort A, et al (2011) Scikit-learn: Machine learning in Python. Journal of Machine Learning Research 12:2825–2830

[5] Nadeau C, Bengio Y (1999) Inference for the generalization error. Advances in Neural Information Processing Systems 12

[6] Gao C, Shinkareva SV, Desai RH (2022) Scope: The South Carolina psycholinguistic metabase. Behavior Research Methods pp 1–32. URL https://doi.org/10.3758/s13428-022-01934-0

[7] Warriner AB, Kuperman V, Brysbaert M (2013) Norms of valence, arousal, and dominance for 13,915 English lemmas. Behavior Research Methods 45:1191–1207. URL https://doi.org/10.3758/s13428-012-0314-x

[8] Mohammad S, Turney P (2010) Emotions evoked by common words and phrases: Using mechanical turk to create an emotion lexicon. In: Proceedings of the NAACL HLT 2010 Workshop on Computational Approaches to Analysis and Generation of Emotion in Text, pp 26–34, URL https://doi.org/10.1111/j.1467-8640.2012.00460.x

[9] Scott GG, Keitel A, Becirspahic M, et al (2019) The Glasgow norms: Ratings of 5,500 words on nine scales. Behavior Research Methods 51(3):1258–1270. URL

https://doi.org/10.3758/s13428-018-1099-3

[10] Brysbaert M, Warriner AB, Kuperman V (2014) Concreteness ratings for 40 thousand generally known english word lemmas. Behavior Research Methods 46:904–911. URL https://doi.org/10.3758/s13428-013-0403-5

[11] Brysbaert M (2017) Age of acquisition ratings score better on criterion validity than frequency trajectory or ratings "corrected" for frequency. Quarterly Journal of Experimental Psychology 70(7):1129–1139. URL https://doi.org/10.1080/17470218.2016.1172097

[12] Brysbaert M, New B (2009) Moving beyond Kučera and Francis: A critical evaluation of current word frequency norms and the introduction of a new and improved word frequency measure for American English. Behavior Research Methods 41(4):977–990. URL https://doi.org/10.3758/brm.41.4.977

4 Figure legends

4.1 Figure 1

Investigating the impact of psychometric item ordering on inter-item correlations. **A.** Order 1 inter-item correlations. **B.** Order 2 inter-item correlations. **C.** Order 1 minus order 2 inter-item correlations.

4.2 Figure 2

Investigating the impact of psychometric item ordering on performance. *Psychometric 1* is obtained from participants that received the following order 1 (as listed in text). *Psychometric 2* participants received the reverse order. *Psychometric* is an aggregate of orders 1 and 2 (as used in the main analysis), and *Psychometric 1 & Psychometric*

Table 1

Norm	Category	Description	Source
Valence	Affect	The pleasantness of a stimulus on a 1 (happy) to 9 (unhappy) scale.	[7]
Arousal	Affect	The intensity of emotion provoked by a stimulus on a scale of 1 (calm) to 9 (aroused) scale.	[7]
Dominance	Affect	The degree of control exerted by a stimulus on a scale of 1 (controlled) to 9 (in control) scale.	[7]
Emotional Association Anticipation	Affect	Word-emotion association built by manual annotation using Best-Worst Scaling method, with 0 (not associated) and 1 (associated) ratings for anticipation.	[8]
Emotional Association Fear	Affect	Word-emotion association built by manual annotation using Best-Worst Scaling method, with 0 (not associated) and 1 (associated) ratings for fear.	[8]
Emotional Association Anger	Affect	Word-emotion association built by manual annotation using Best-Worst Scaling method, with 0 (not associated) and 1 (associated) ratings for anger.	[8]
Emotional Association Disgust	Affect	Word-emotion association built by manual annotation using Best-Worst Scaling method, with 0 (not associated) and 1 (associated) ratings for disgust.	[8]
Emotional Association Joy	Affect	Word-emotion association built by manual annotation using Best-Worst Scaling method, with 0 (not associated) and 1 (associated) ratings for joy.	[8]
Emotional Association Trust	Affect	Word-emotion association built by manual annotation using Best-Worst Scaling method, with 0 (not associated) and 1 (associated) ratings for trust.	[8]
Emotional Association Surprise	Affect	Word-emotion association built by manual annotation using Best-Worst Scaling method, with 0 (not associated) and 1 (associated) ratings for surprise.	[8]
Emotional Association Sadness	Affect	Word-emotion association built by manual annotation using Best-Worst Scaling method, with 0 (not associated) and 1 (associated) ratings for sadness.	[8]
Imageability	Concreteness	The degree of effort involved in generating a mental image of the concept on a 1 (unimaginable) to 7 (imageable) scale.	[9]
Concreteness	Concreteness	The degree to which the concept can be experienced directly through the senses from a 1 (abstract) to 5 (concrete) scale.	[10]
Familiarity	Frequency	A word's subjective familiarity on a 1 (unfamiliar) to 7 (familiar) scale.	[9]
Age of Acquisition	Frequency	The age at which people acquired the word, in which a three-choice test was administered to participants in grades 4 to 16 (college) (Living Word Vocabulary Test).	[11]
Frequency	Frequency	Log10 version of frequency norms based on the SUBTLEXus corpus.	[12]

2 is the concatenation of both orderings. Error bars are adjusted 95% confidence intervals [3].

4.3 Figure 3

Evaluating how test performance varies with training set size for 3 data (sub-)sets: (i) Basel Risk Norms (All), which refers to our full data set of 1,004 risks, (ii) Basel Risk Norms (Bhatia Set), referring to our data limited to the same 306 risks as used in [2], and [2] (iii). Test sets are composed of all remaining risks in the data. Train–test splits were sampled randomly (i.e., bootstrapped), with 10 repetitions per model per training set size. Error bars are 95% confidence intervals.

4.4 Figure 4

Pairwise differences between elastic net and gradient boosting using 10x10-fold cross-validation. Cross-validation via [2]'s risk norms (306 risks) are colored cyan and points obtained using the Basel Risk Norms (1004 risks) are colored purple. Error bars are adjusted 95% confidence intervals [3].

4.5 Figure 5

Pairwise differences between standardized and unstandardized models using 10x10-fold cross-validation and elastic net regression. Cross-validation via [2]'s risk norms (306 risks) are colored cyan and points obtained using the Basel Risk Norms (1004 risks) are colored purple. Error bars are adjusted 95% confidence intervals [3].

4.6 Figure 6

Heatmap illustrating the differences in 10x10-fold cross-validation R-squared between all pairwise model combinations using elastic net regression (y-axis models minus x-axis models). White squares reflect mean differences that do not significantly differ from zero. The top panel shows the results for the data of [2] and the bottom panel the results for the Basel Risk Norms.

5 Table Legends

5.1 Table 1

Word norms and their sources.

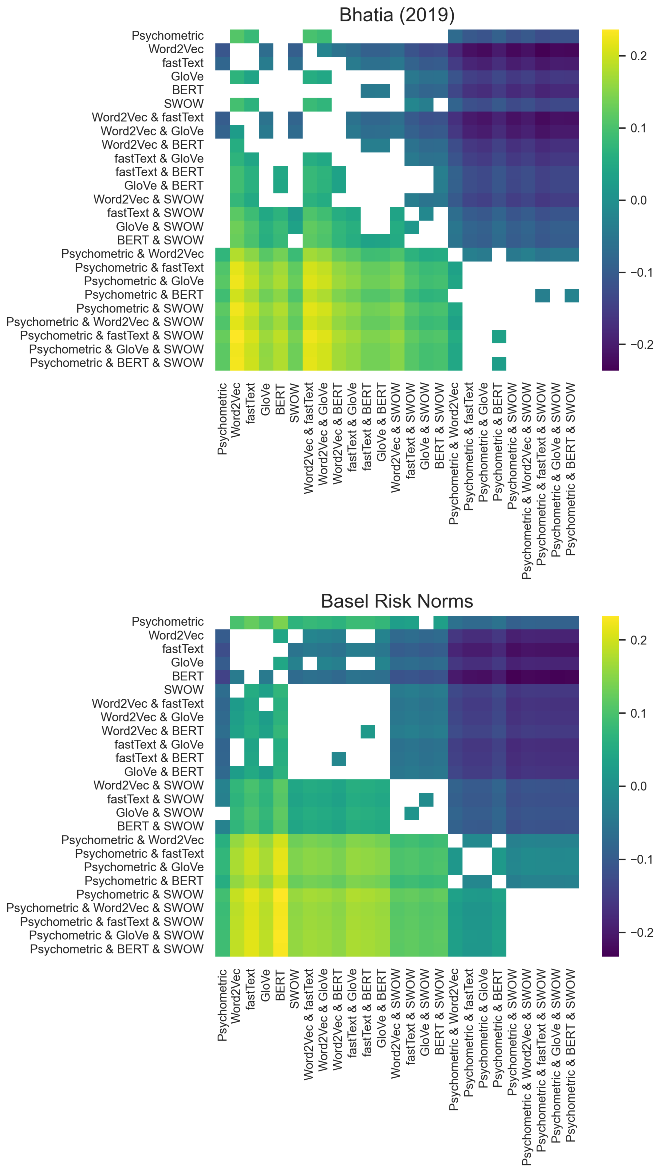

Fig. 6 Heatmap illustrating the differences in 10x10-fold cross-validation R-squared between all pairwise model combinations using elastic net regression (y-axis models minus x-axis models). White squares reflect mean differences that do not significantly differ from zero. The top panel shows the results for the data of [2] and the bottom panel the results for the Basel Risk Norms.
